# TOP2A promotes proliferation and metastasis of hepatocellular carcinoma regulated by miR-144-3p

**DOI:** 10.7150/jca.64017

**Published:** 2022-01-01

**Authors:** Tao Wang, Jing Lu, Rangrang Wang, Wanyue Cao, Junming Xu

**Affiliations:** 1 Department of General Surgery, Shanghai General Hospital, School of Medicine, Shanghai Jiaotong University, Shanghai, 200080, China.; 2 Department of General Surgery, Shanghai East Hospital, Tongji University School of Medicine, Shanghai, 200120, China.

**Keywords:** TOP2A, miR-144-3p, cell cycle, EMT, Hepatocellular carcinoma

## Abstract

Topoisomerase II alpha (TOP2A) is an important nuclear protein which is found in various types of cancers. Whether TOP2A plays an important role in hepatocellular carcinoma (HCC) remains unclear. Through bioinformatic analysis and clinical specimen verification, we found that TOP2A is highly expressed in HCC and is associated with poor prognosis. Knockdown and overexpression of TOP2A can respectively inhibit or promote proliferation, metastasis and invasion of HCC cells *in vitro* and *in vivo*. Mechanismly, TOP2A activates cell cycle progression from G2 to M phase by inhibiting the phosphorylation of CHK1 and promotes Epithelial-to-mesenchymal transition (EMT) process. We further confirmed that TOP2A is a direct target of miR-144-3p whose overexpressing can partially reverse the effect of TOP2A in HCC cells. Our data suggested that TOP2A functions by promoting the proliferation, migration, invasion and EMT process of HCC and can be considered as a potential target for the treatment of HCC.

## Introduction

Liver cancer is world-widely the fourth cause of death caused by cancers, among which hepatocellular carcinoma accounts for the majority 1. Overall prognosis for HCC patients remains poor due to the insidious onset and aggressive biological features of HCC 2, 3, which leads to advanced clinical stages and high recurrence rate of HCC patients 4. Although surgical resection for early-stage HCC brings good outcomes 5, a large percentage of HCC are only found in the late stage because the tumor is unresectable and the prognosis is poor 6. Therefore, it is very significant to search for new biomarkers and targets for the drug design of HCC.

TOP2A is one isoform of the Topoisomerase II (TOP2) which is a nuclear protein required for DNA replication and cell division. TOP2A is highly expressed in proliferating and growing cells. It is also detected in diverse human malignancies such as adrenocortical, nasopharyngeal, primary breast cancer and colon cancer 7-11. A variety of TOP2A inhibitors which target TOP2A have been applied in promising treatment for cancers 12, 13. It is understood that TOP2A is abnormally expressed in HCC, but the underlying mechanism remains to be elucidated 14.

MicroRNA (miRNA) is small non-coding RNA which inhibits the translation and stability of messenger RNA (mRNA). miRNA plays roles in inflammation, stress response, differentiation, cell cycle regulation, apoptosis and migration^15^. In studies of miRNA and cancer, the theme that the expression of miRNA in malignant cells varies significantly compared with that in normal cells is widely discussed. miRNA can be hallmarks of cancers^16^. Furthermore, miRNA is considered as oncogenes or tumor suppressor genes respectively 15. For example, studies showed that miR-218 and miR-200 prevent the proliferation and metastasis of HCC cells whereas miR-330 and miR-517a promote the development of HCC 17-20. Other studies reported that miR-144-3p functions as a key suppressor of HCC but the underlying mechanism is to be further identified 21.

In our study, we found that TOP2A was not only upregulated in both HCC cells and tissues, but also closely associated with the prognosis and clinicopathological characteristics of HCC patients. Furthermore, we demonstrated that TOP2A promoted proliferation and metastasis of HCC cells regulated by miR-144-3p. On the one hand, TOP2A inhibits the phosphorylation of CHK1, leading to the cell cycle transition from G2 to M phase. On the other hand, TOP2A promotes the metastasis of hepatocellular carcinoma by regulating the EMT process. Together, our results indicate that the overexpression of TOP2A regulated by miR-144-3p facilitates HCC proliferation and metastasis, and thus we propose that TOP2A can be considered as a potential target for the treatment of HCC.

## Material and Methods

### HCC Datasets and clinical specimen collection

The Cancer Genome Atlas Liver Hepatocellular Carcinoma (TCGA-LIHC) and corresponding clinical data used in this study were downloaded from TCGA. The 90 pairs of HCC and adjacent normal tissues analyzed in this study were collected from patients at Shanghai General Hospital between January, 2009 and July, 2014. The study population was selected according to the following criteria.

The inclusion criteria: I) pathological diagnosed with HCC; II) follow-up data was available. The exclusion criteria: I) incomplete patient information; II) with a history of other tumor diseases; III) received immunotherapy, chemotherapy, radiotherapy or any other related anti-tumor therapies before surgeries.

The surgically removed tissues were immediately frozen in the -80℃ refrigerator for subsequent protein and RNA extraction. This research was approved by the Ethics Committee of Shanghai General Hospital and informed consents were obtained from all patients enrolled in the study.

### Cell culture and stable cell line construction

The hepatocellular carcinoma cell lines (MHCC97-H, Huh-7, LM3, Hep-G2, Bel-7402, and Hep-3B) and normal LO2 cell lines were obtained from the Shanghai Cell Bank of the Chinese Academy of Sciences (Shanghai, China). Cell culture was performed using Dulbecco's modified Eagle's medium (Gibco, USA) containing 10% fetal bovine serum and 1% penicillin streptomycin (Gibco, USA). Cells grew under a moist atmosphere containing 5% CO_2_ at 37°C. Lentiviral preparations were generated by transiently transfecting HEK-293 T cells with psPAX2 and pMD2.G plasmids using Lipofectamine 2000 according to the manufacturer's protocol. Negative control (NC) and empty vector (EV) were used as controls. MiR-144-3p mimics and inhibitors were purchased from GenePharma.

### Real-time quantitative PCR (RT-qPCR)

TRIzol (Takara Biotechnology, Japan) was used to extract total RNA from the tissue samples and HCC cell lines according to the manufacturer's instruction, followed by applying a reverse transcription kit (Takara Biotechnology, Japan) in synthesizing cDNA for subsequent PCR assay. Using cDNA as template, we performed RT-qPCR with SYBR Premix Ex Taq II (Takara, Shiga, Japan). The relative mRNA expression levels were normalized to GAPDH and calculated by the 2^-ΔΔct^ method. All samples were analyzed in three replicates. The primers are shown in **Table S1**.

### Western Blot

Total protein was extracted from the tissue samples and cells using RIPA and phenylmethyl sulfonyl fluoride (PMSF). Protein concentrations were quantified using a BCA protein kit (Yeasen, Shanghai, China). Equivalent proteins were separated by SDS-PAGE and transferred to PVDF membranes (Millipore, Billerica, MA, USA). The membranes were blocked using 5% skim milk-TBST and incubated with primary antibodies at 4 °C overnight. The membranes were washed for 30 minutes, 10 minutes at a time in TBST, and incubated with secondary antibodies at room temperature for 2 hours. ECL chemiluminescence is conducted to detect protein signals. GAPDH was chosen as the internal reference protein. Antibodies against the following proteins were used: GAPDH (5174, CST), TOP2A (12286, CST and A4389, ABclonal), CHK1 (ab40866, Abcam), p-CHK1 (ab278717, Abcam), CDK1 (ab133327, Abcam), Cyclin B1 (ab32053, Abcam), E-Cadherin (3195, CST), N-Cadherin (13116, CST), Vimentin (5741, CST) and Slug (9585, CST).

### Immunohistochemistry (IHC)

The sections were baked at 56°C for 2 hours for dewaxing, boiled in citrate buffer for antigen retrieval, and blocked using 3% hydrogen peroxide. The contents were incubated with the primary antibody against TOP2A (1:25, 12286, CST) at 4°C overnight and biotinylated with a goat anti-rabbit secondary antibody for 1 hour. At last, the reaction was visualized using DAB, and the sections were counterstained with hematoxylin. IHC scores were calculated by multiplying the percentage and intensity score of stained cells (staining intensity: negative = 0, weak = 1, moderate = 2, strong = 3, and staining extent: 0 = no staining, 1 = 0%-25%, 2 = 25%- 50%, 3 = 50%-75% and 4 = 75%-100%). The total score was calculated as intensity score × extent score.

### CCK-8 assay

Cell viability was measured using the Cell Counting Kit-8 (CCK-8) assay (NCM Biotech, Suzhou, China) to evaluate cell proliferation. Cells were seeded into 96-well plates at 2000 cells per well. The absorbance at 450 nm was measured with a spectrophotometer at different time points (0, 24, 48, 72 and 96 h).

### Colony formation assay

Transfected cells and control cells were seeded into 6-well plates at 1000 cells per well. After 2 weeks of culture, the cells were washed three times using PBS, fixed with 4% paraformaldehyde and stained with crystal violet solution.

### Scratch wound healing assay

Cells were seeded into 6-well plates and cultured to 85% confluence. The cell layers were scratched using a sterile 200μL pipette tip and then washed three times with PBS to remove the scratched cells. Then, the remaining cells were cultured in serum free DMEM (Gibco, USA). Cells were observed using an inverted microscope and photographed at 0 and 48h respectively.

### Transwell assay

Transwell assay was conducted for cell migration and invasion studies. Cells in 200μL serum-free medium were seeded into the upper chamber, and 600μL DMEM containing 10% FBS (Gibco, USA) was added to the lower chamber. Matrigel (Corning, NY, USA) was added to precoat the upper chamber before cell seeding only for cell invasion study. The cells were fixed after 24h of culture. Cells on the underside of the membrane were stained with 0.1% crystal violet and counted under a microscope.

### Flow cytometry and EdU assay

Cells were fixed in 70% ethanol and kept at 4°C for 30 minutes. Cell suspension was stained with propidium iodide and analyzed by FACS according to the manufacturer's instruction. Cells were seeded into 12-well plates and incubated with the Cell-Light EdU Apollo 567 (catalog No. C10310-1; RiboBio) for 2h. Nucleosides were removed after incubation by three wash steps with PBS.

### *In vivo* assays

10^6^ cells were injected subcutaneously into the right lower abdomen of 6-week-old male nude mice (Shanghai SLAC Laboratory Animal Co., Ltd., Shanghai, China) to build a tumor-bearing model (five mice per group). We used Vernier calipers to measure the tumor size every 5 days. After 4 weeks, all mice were euthanized to collect and fix the tumors in formalin. Tumor volume was calculated using the formula: Volume=Length*Width^2^*0.5. Furthermore, IHC was used to detect the Ki-67 protein expression in xenograft tumors. 2*10^5^ cells were injected into the tail vein of nude mice to establish lung metastasis tumor model.

### Dual luciferase reporter assay

The bioinformatic analysis website TargetScan dataset was applied and we found that TOP2A may be a potential target of miRNA. 100 ng of wild type (WT) or mutation (MUT) 3′-UTR of TOP2A vector was transfected into HCC cells using the Lipofectamine 2000 reagent (Invitrogen, USA) according to the manufacturer's protocol. After 48 hours, Renilla luciferase activity was detected using a Dual-Luciferase Reporter Assay System (Promega). Firefly luciferase was normalized to the Renilla signal.

### Statistical analysis

The relationships between TOP2A and clinicopathological features were analyzed by Wilcoxon signed rank sum test and logistic regression. Cox regression analysis and the Kaplan-Meier method were applied to analyze the relationships of the clinicopathological features with overall survival according to the data from the TCGA database. The boundary value of TOP2A expression was determined by its mean value. In TMA, the boundary value of TOP2A expression was determined by its mean IHC score. The data are presented as Mean ± SD. Student's t-test was used for comparison between two groups, and one-way ANOVA followed by the least square difference (LSD) tests was performed for comparison among multiple groups. P < 0.05 indicated a significant difference.

## Results

### TOP2A is upregulated in HCC tissues

**Figure 1:**
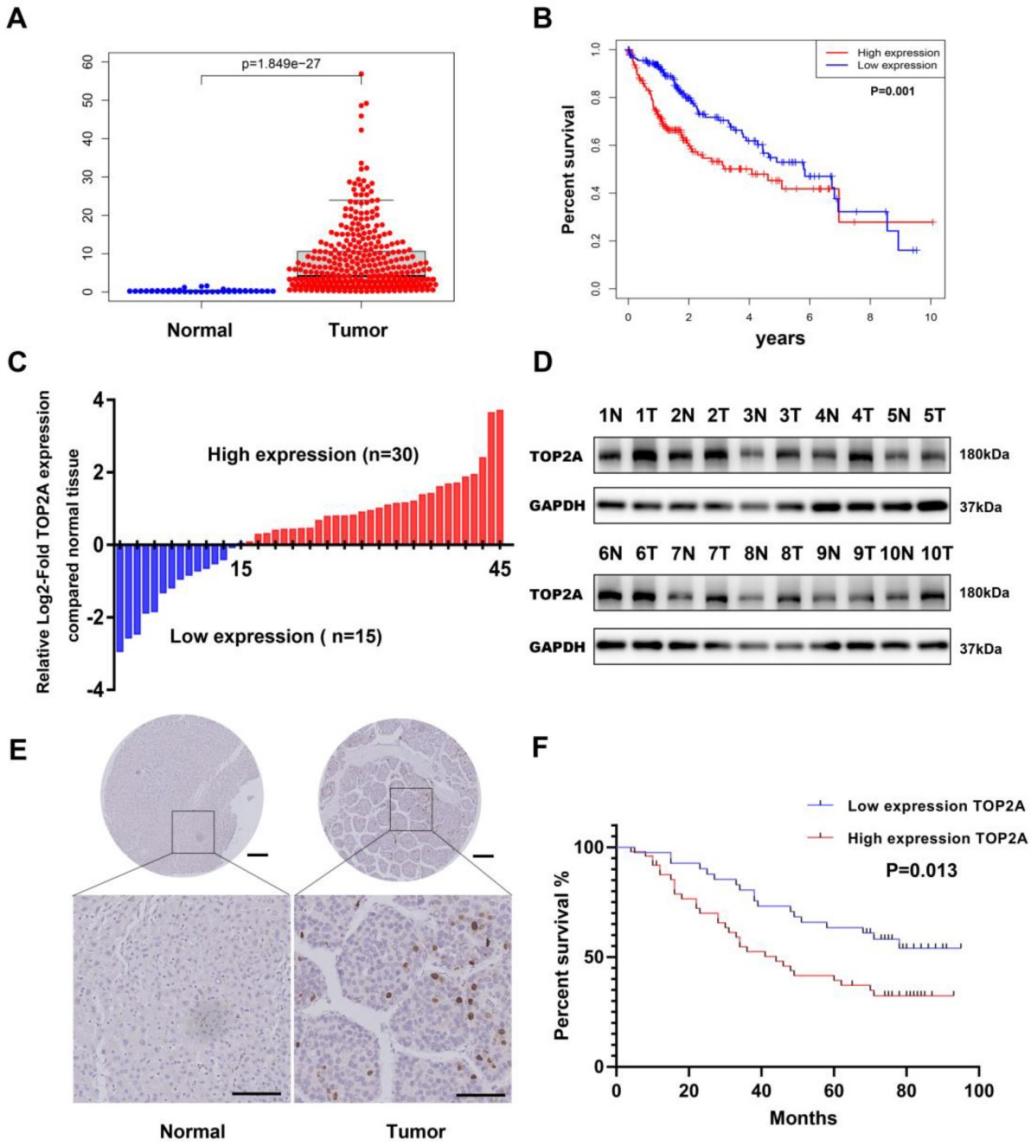
** TOP2A is upregulated in HCC and associated with poor prognosis. (A)** mRNA expression level of TOP2A in HCC tissues and normal tissues from the TCGA database. **(B)** The relationship between TOP2A expression and overall survival of HCC patients according to the data obtained from TCGA-LIHC cohort. **(C)** RT-qPCR detected the mRNA expression of TOP2A in 45 paired HCC tissues and adjacent normal tissues. **(D)** Western blot detected the protein expression of TOP2A in 10 representative pairs of HCC tissues and adjacent nontumor tissues.** (E)** IHC staining scores of TOP2A expression in 90 paired HCC tissues and adjacent normal tissues. Representative images of different TOP2A expression levels are shown. up panel, scale bar 200μm; down panel, scale bar 100μm. **(F)** The relationship between TOP2A expression and overall survival of HCC patients according to the data obtained from TMA.

To investigate the expression level of TOP2A, we first downloaded the gene expression data of HCC tissue from TCGA (The Cancer Genome Atlas) database and analyzed the mRNA expression level of TOP2A. Compared with normal tissues, the expression level of TOP2A in HCC tissues significantly increased (**Figure 1A**). Moreover, the expression of TOP2A in HCC was higher than that of non-tumor tissues according to the ONCOMINE database (**Figure S1A**). Based on the medium value of TOP2A expression, the patients were divided into a high-expression group and a low-expression group, the results of Kaplan-Meier analysis showed that the survival rate of high expression group was lower than that of low expression group (**Figure 1B**). To verify the results of the abnormality, we conducted RT-qPCR to analyze the TOP2A mRNA level in 45 pairs of HCC and matched adjacent normal tissues from Shanghai General Hospital. We found that the mRNA of TOP2A was highly expressed in 30/45(66.7%) of the HCC tissues compared with the adjacent normal tissues (**Figure 1C**). In addition, the results of Western blot showed that the protein expression levels of TOP2A were also upregulated in HCC tissues compared with adjacent tissues (**Figure 1D**). Furthermore, IHC staining analysis was used to detect the TOP2A protein expression level in tissue microarray (TMA) which contains 90 pairs of HCC and matched adjacent normal tissues. The results revealed that the protein expression level of TOP2A in HCC tissues was higher than that in adjacent normal tissues (**Figure 1E**). In summary, TOP2A was found to be upregulated in HCC tissues in both the mRNA and protein levels.

### TOP2A is related to clinical characteristics and poor prognosis of HCC

We analyzed the correlation between the TOP2A expression level and the clinical characteristics of HCC patients by HCC TMA. 90 patients were separately divided into TOP2A high expression group and low expression group by IHC scores. The results showed that high levels of TOP2A expression were positively correlated with tumor grade, tumor T stage, tumor M stage and tumor TNM stage, while there was no correlation between TOP2A expression and gender, age or tumor N stage (**Table 1**). Kaplan-Meier survival analysis suggested that HCC patients with high TOP2A expression had worse prognosis than those with low TOP2A expression (**Figure 1F**).

### TOP2A promotes HCC cells proliferation, migration and invasion *in vitro*

**Figure 2:**
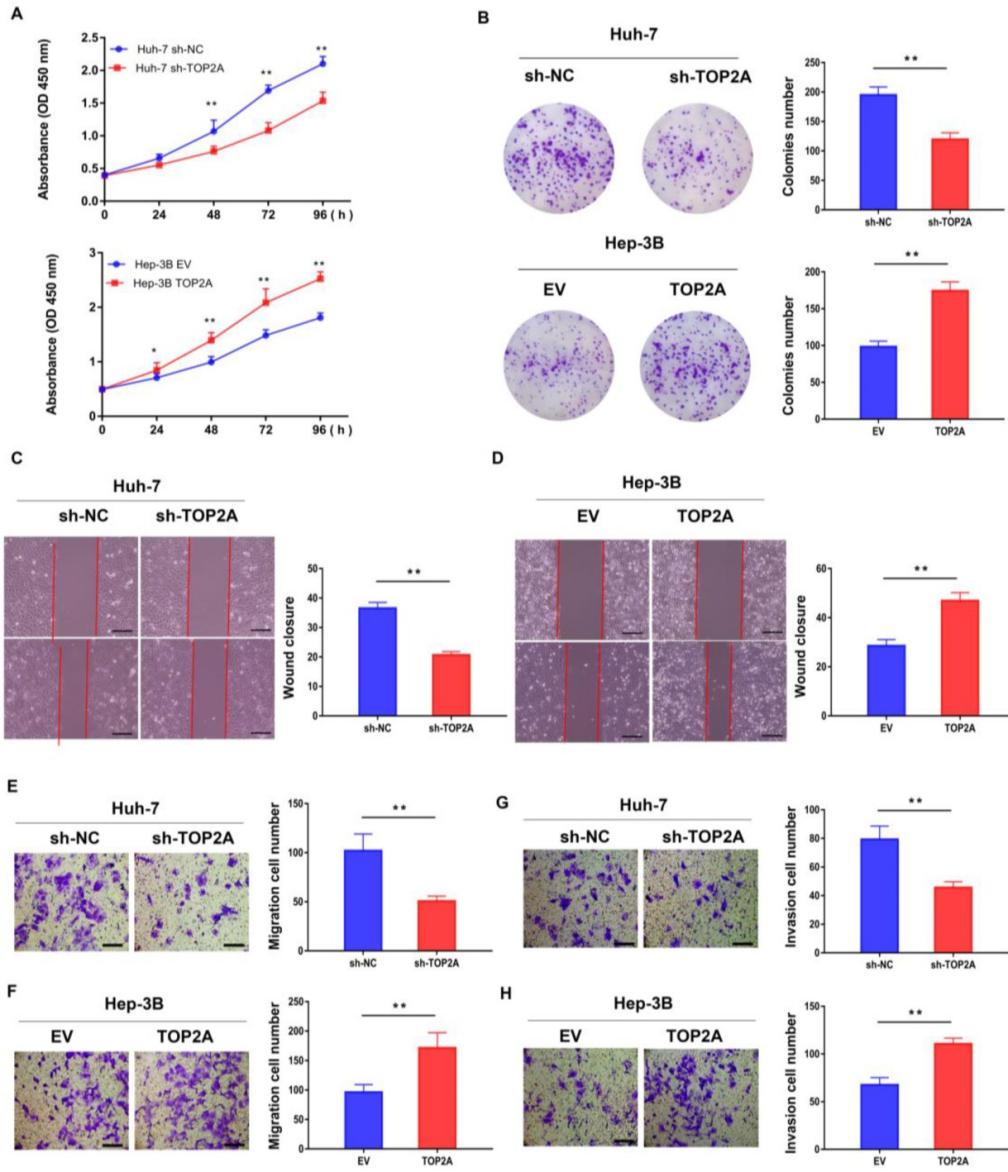
** TOP2A promotes HCC cells proliferation, migration and invasion *in vitro.* (A)** CCK-8 assays were performed in HCC cells with different levels of TOP2A expression. **(B)** Colony formation experiments performed in HCC cells with different levels of TOP2A expression. **(C, D)** Scratch wound assays performed in HCC cells with different levels of TOP2A expression, scale bar 200μm. **(E, F)** Transwell migration assays performed in HCC cells with different levels of TOP2A expression, scale bar 200μm. **(G, H)** Transwell invasion assays performed in HCC cells with different levels of TOP2A expression. Scale bar 200μm. *P < 0.05, ** *P* < 0.01.

RT-qPCR and Western blot were used to detect both the mRNA and protein expression of TOP2A in six human hepatoma and normal LO2 cell lines (MHCC97-H, Huh-7, LM3, HepG2, SMMC-7402, Hep-3B and LO2). Among these HCC cell lines, Huh-7 and Hep-3B cells showed the highest and lowest TOP2A expression levels (**Figure S1B and S1C**), according to which Huh-7 and Hep-3B cell lines were selected to establish stable knockdown (Huh-7) and overexpression (Hep-3B) cell lines respectively (**Figure S1D-S1F**). CCK-8 assay revealed that the cell viability of Huh-7 cells with TOP2A knockdown was decreased and Hep-3B cells with TOP2A overexpression were increased compared with the levels observed in the control group (**Figure 2A**). Colony formation experiments were carried out and the results showed that the number of colonies with TOP2A knockdown in Huh-7 was decreased significantly. In contrast, Hep-3B cells overexpressing TOP2A yielded opposite results (**Figure 2B**). Scratch wound assays showed that knocking down TOP2A can prohibit wound closure whereas overexpressing TOP2A promoted wound closure compared to the control groups. (**Figure 2C and 2D**). Transwell migration and invasion assays were conducted to assess cell migration and invasion abilities. The results showed that knockdown or overexpression of TOP2A dramatically decreased or increased the migration and invasion rates respectively, compared with the controls (**Figure 2E-2H**).

### TOP2A enhances tumorigenesis and metastasis of HCC* in vivo*

**Figure 3:**
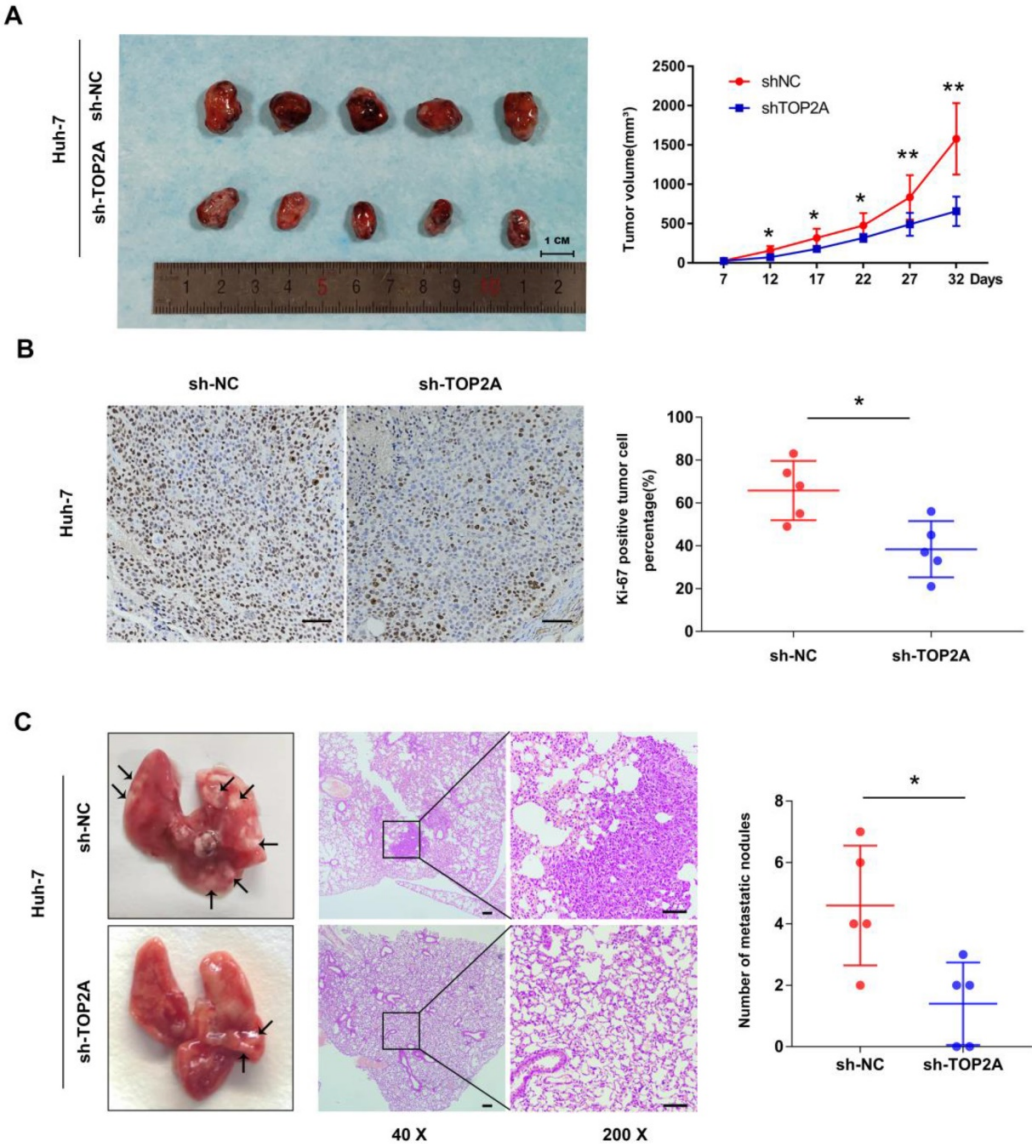
** TOP2A enhances tumorigenesis and metastasis of HCC *in vivo.* (A)** Images of nude mice bearing subcutaneous tumor xenografts derived either from sh-TOP2A or Negative Control cells and volume of tumors derived from sh-NC and sh-TOP2A Huh-7 cells. **(B)** IHC staining of Ki-67 in xenograft tumor tissue derived from sh-NC and sh-TOP2A Huh-7 cells, scale bar 100μm. **(C)** Representative images and HE images of lung metastatic nodules derived from sh-NC and sh-TOP2A Huh-7 cells, left panel, scale bar 200μm; right panel, scale bar 100μm. *P < 0.05, **P < 0.01.

The tumorigenesis ability of cancer cells *in vivo* was detected by a subcutaneous xenograft model. As shown in** Figure 3A**, the tumor volume collected from Huh-7 cells with TOP2A knockdown was smaller than that of the controls. Ki-67 protein expression was down-regulated in xenograft tumors in which TOP2A was knocked down, compared with the controls according to IHC (**Figure 3B**). In order to extend our understanding on the effects of TOP2A on tumor metastasis *in vivo*, we injected Huh-7 cells into tail veins of nude mice. After four weeks, the TOP2A knockdown groups had fewer metastatic nodules on lung surfaces whereas TOP2A overexpression groups showed more metastatic nodules on lung surfaces (**Figure 3C**). We also injected Hep-3B cells into subcutaneous and tail veins of nude mice, but we did not observe the formation of subcutaneous tumor and lung metastatic nodules. These results implied that TOP2A enhances the tumorigenesis and metastasis capability of HCC *in vivo.*

### TOP2A activates cell cycle progression from G2 to M phase and promotes EMT process

**Figure 4:**
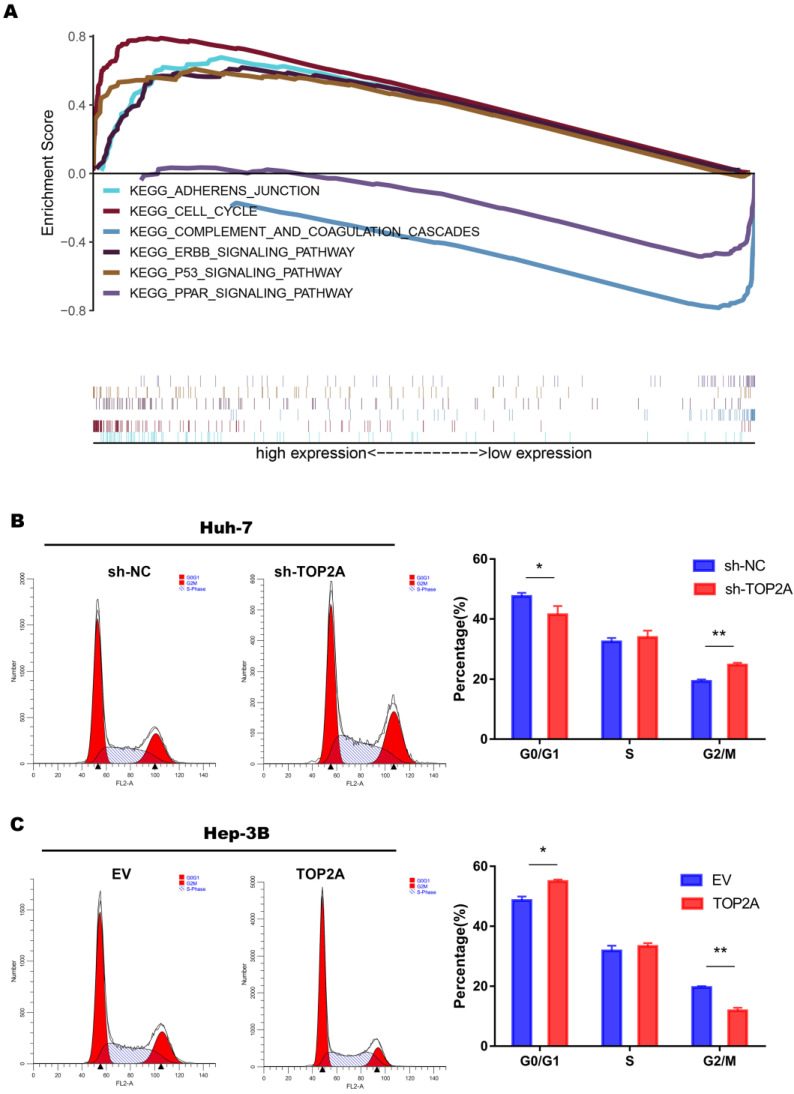
** TOP2A activates cell cycle progression from G2 to M phase. (A)** The result of gene set enrichment analysis (GSEA) according to the data obtained from TCGA database. **(B, C)** Analysis of flow cytometry for cell cycle detection in HCC cells with different levels of TOP2A expression.

**Figure 5:**
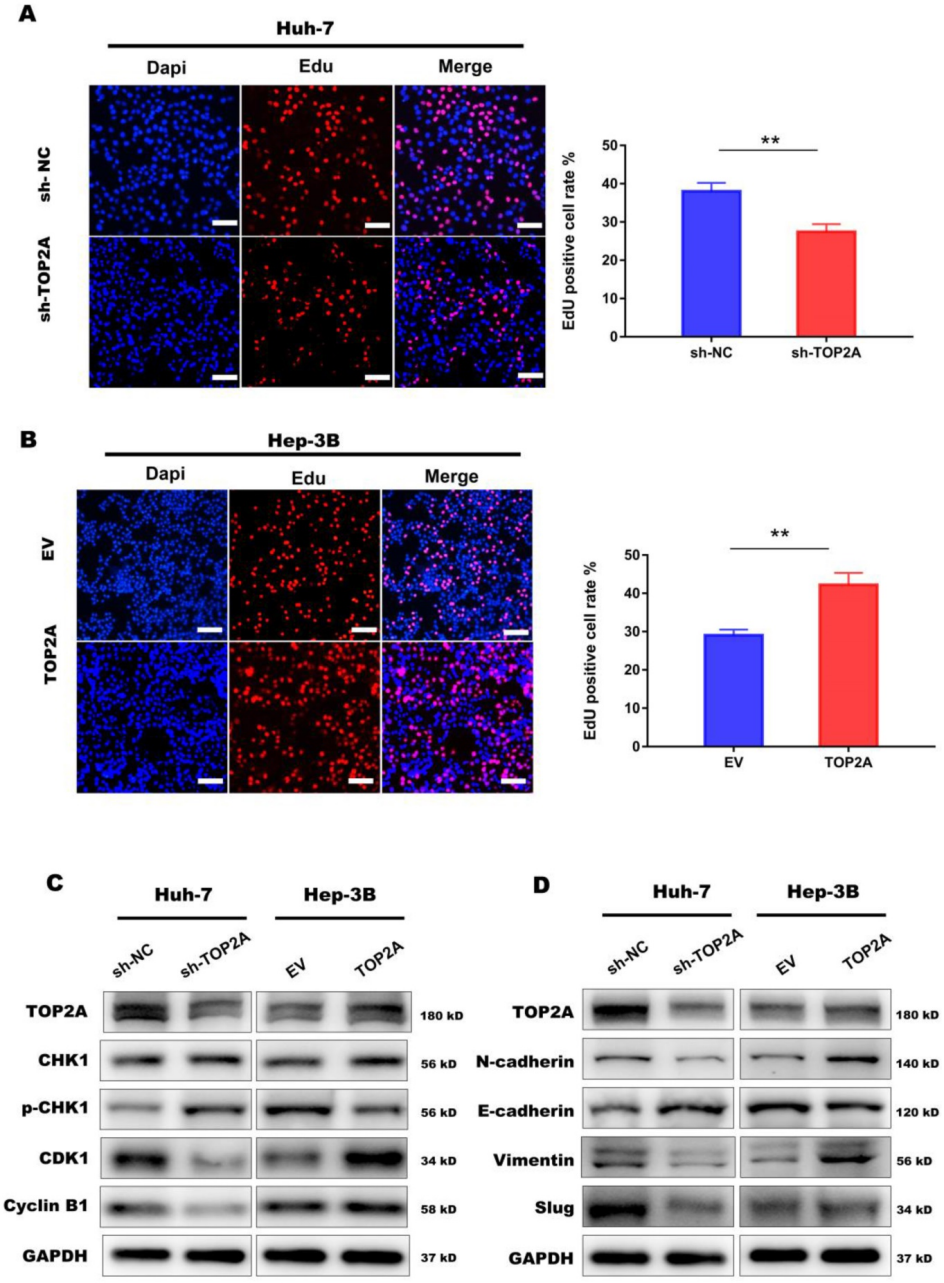
** TOP2A activates cell cycle progression and promotes EMT process. (A, B)** EdU assay performed in HCC cells with different levels of TOP2A expression. Scale bar 200μm. **(C)** G2/M checkpoint related proteins were detected by Western blot. **(D)** EMT related proteins were detected by Western blot. *P < 0.05, ** *P* < 0.01.

In order to explore the specific mechanism of HCC proliferation promoted by TOP2A, we first identified patients with low or high TOP2A expression from TCGA database and performed gene set enrichment analysis (GSEA). The result showed that the cell cycle pathway was highly correlated to patients with high expression of TOP2A (**Figure 4A**). Fluorescence-activated cell sorting showed that TOP2A knockdown in Huh-7 cells caused an increase in the percentage of G2, M-phase cells while the percentage of G1-phase cells was decreased at the same time. TOP2A overexpression in Hep-3B led to the opposite results (**Figure 4B and 4C**). Furthermore, we used EdU assay to verify whether TOP2A knockdown or overexpression resulted in the proliferation of Huh-7 and Hep-3B cells. Fluorescence was reduced in Huh-7 cells with TOP2A knockdown while it increased in Hep-3B cells with TOP2A overexpression (**Figure 5A and 5B**). Results from Western blot performed in cycle-related protein experiments demonstrated that the knockdown of TOP2A increased the protein level of phosphorylated CHK1 (p-CHK1) in Huh-7 cells but did not affect total CHK1 expression. Conversely, overexpression of TOP2A decreased the level of p-CHK1 protein in Hep3B cells. The expression levels of CDK1, the downstream protein of CHK1, and Cyclin B were decreased in the TOP2A knockdown model. As for Hep-3B cells, the expression of CHK1 was not affected and the expression of p-CHK1 was decreased. On the contrary, CDK1 and Cyclin B protein expression were increased (**Figure 5C**).

At the same time, we noticed that cell-cell adherent junctions (AJS) were also significantly related to the high expression of TOP2A according to GSEA result (**Figure 4A**). Numbers of studies have reported that EMT and AJS are closely related 22, 23. We then investigated the expression of EMT markers, such as N-cadherin, E-cadherin, Vimentin and Slug, using Western Blot in TOP2A knockdown cells. The results showed that epithelial molecules E-cadherin were downregulated while mesenchymal molecules, N-cadherin, Vimentin and Slug were significantly upregulated compared to negative controls (**Figure 5D**).

These findings indicated that TOP2A activates cell cycle progression from G2 to M phase by inhibiting the phosphorylation of CHK1 and promotes EMT process.

### TOP2A is a direct target of miR-144-3p

**Figure 6:**
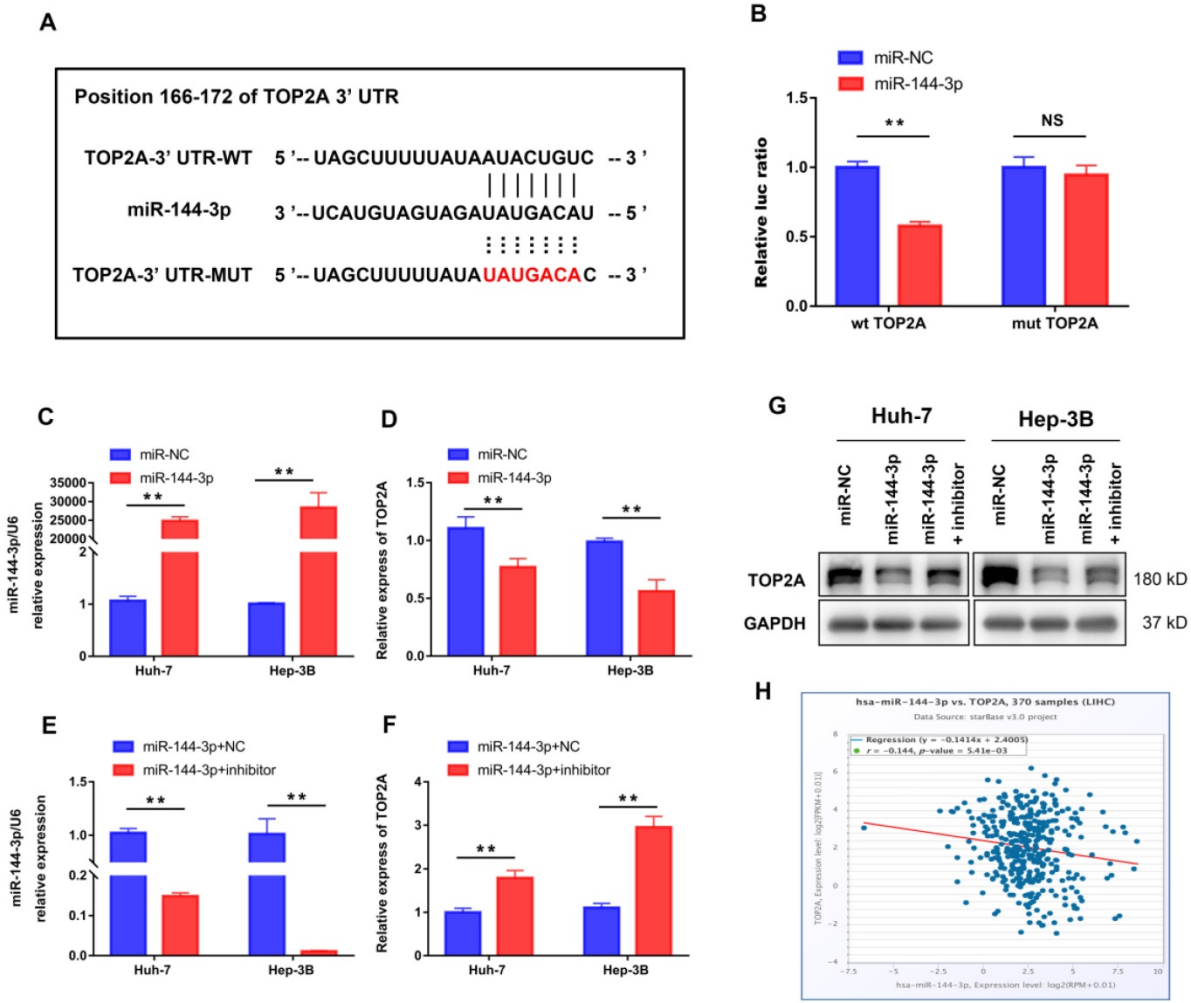
** TOP2A is a direct target of miR-144-3p. (A)** miR-144-3p and its putative binding sequence in the wild-type and mutant 3′-UTR of TOP2A. **(B)** Overexpression of miR-144-3p significantly decreased the luciferase activity of constructs with negative control but not mutant (MUT) TOP2A 3′-UTR. **(C, D)** RT-qPCR showed that mRNA of TOP2A was significantly decreased in HCC cells using miR-144-3p mimics. **(E, F)** RT-qPCR showed that attenuating miR-144-3p expression rescued the suppression of TOP2A by miR-144-3p. **(G)** Western blot showed that attenuating miR-144-3p expression rescued the suppression of TOP2A by miR-144-3p. **(H)** The expression of miR-144-3p and TOP2A in ENCORI database was negatively correlated. ** *P* < 0.01, NS: No Significance.

We further performed bioinformatic analysis using the TargetScan dataset to explore the molecular mechanism of HCC progression by TOP2A and found that TOP2A was a potential target of miR-144-3p (**Figure 6A**). The result from luciferase assay which was applied to study the bounding of miR-144-3p to the 3′-UTR of TOP2A showed significantly weakened luciferase activity compared to negative controls (**Figure 6B**). Then we overexpressed miR-144-3p using miR-144-3p mimics in Huh-7 and Hep-3B cells (**Figure 6C**). The corresponding TOP2A mRNA expression significantly decreased (**Figure 6D**). In addition, attenuating miR-144-3p expression rescued the suppression of TOP2A by miR-144-3p (**Figure 6E and 6F**), which was also approved by the result of Western Blot (**Figure 6G**). Furthermore, we collected gene expression data in HCC tissues from ENCORI (http://starbase.sysu.edu.cn/) and found that the expression of miR-144-3p and TOP2A was negatively correlated (**Figure 6H**). These results indicated that TOP2A was a direct downstream target of miR-144-3p in HCC cells.

### Activation of miR-144-3p partially reverses the effect of TOP2A in HCC cells

**Figure 7:**
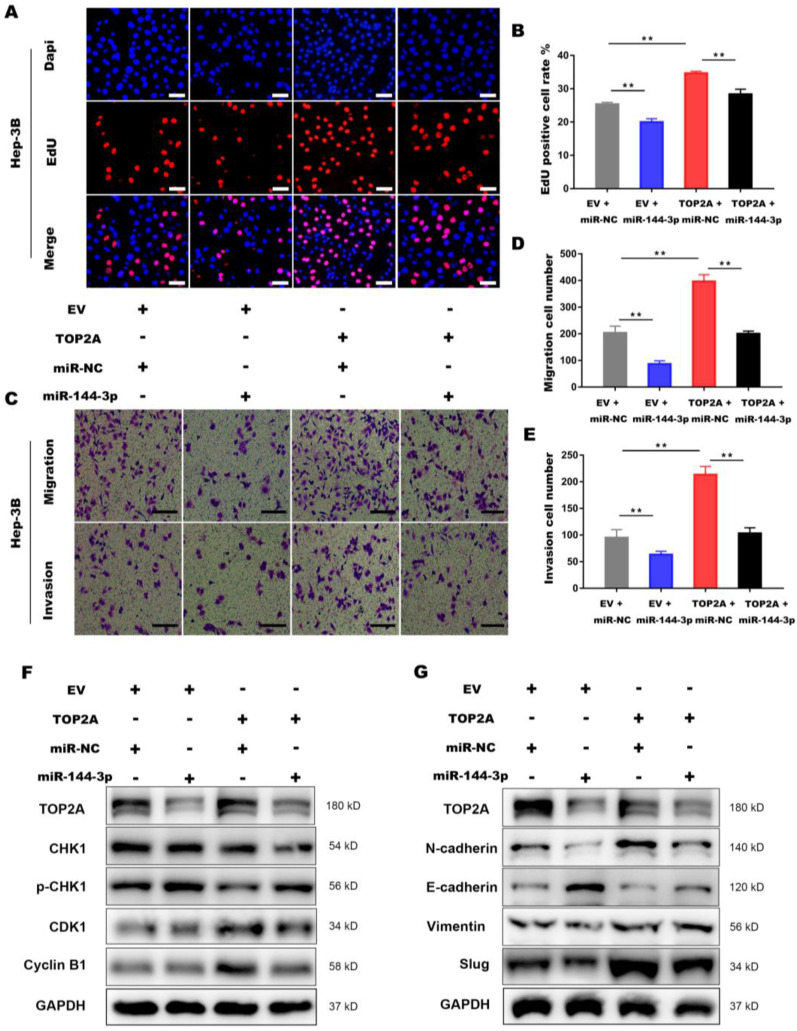
** Activation of miR-144-3p partially reverses the effect of TOP2A in HCC cells. (A, B)** EdU assays showed that the effect of TOP2A on the proliferation in HCC cells was reversed by miR-144-3p. Scale bar 50μm. **(C, D, E)** Transwell migration and invasion assays showed that the effect of TOP2A on the migration, invasion in HCC cells was reversed by miR-144-3p. Scale bar 200μm. **(F, G)** Western blot showed that the expression levels of G2/M check point related proteins and EMT related proteins were reversed by miR-144-3p. ** *P* < 0.01.

To further illustrate the interaction between miR-144-3p and TOP2A, we transfected Hep-3B cells overexpressing TOP2A with miR-144-3p or miR-NC. EdU assays demonstrated that miR-144-3p significantly inhibited TOP2A overexpression, followed by the reversed effect of TOP2A on the proliferation in HCC cells (**Figure 7A and 7B**). The functions of miR-144-3p on HCC cell migration and invasion were identified by transwell chamber assays, revealing that miR-144-3p significantly reduced the HCC cells migration and invasion by TOP2A overexpression (**Figure 7C-7E**). Consistent with this result, the expression level of p-CHK1, CDK1 and Cyclin-B1 were also partially reversed by miR-144-3p (**Figure 7F**) and TOP2A-promoted EMT was also attenuated by miR-144-3p overexpression (**Figure 7G**). Collectively, the above findings suggest that as the upstream regulator of TOP2A, miR-144-3p can partially reverse the effect of TOP2A in HCC cells.

## Discussion

The American Cancer Society estimated that the number of new cases of liver cancer in the United States in 2019 reached 42,030, among which 31,780 deaths occurred due to liver cancer 24. Although clinical comprehensive therapies, including sorafenib and immune checkpoint inhibitors, are used in the treatment of liver cancer, the 5-year survival rate of liver cancer is still less than 20% 25, 26. Therefore, the mechanism of the malignant biological feature of HCC requires further studies. TOP2A, a member of DNA topoisomerase, has been reported to be involved in the tumorigenesis and progression of many tumors 27-30. High expression of TOP2A had effects on poor prognosis and enhanced metastasis capabilities of in pancreatic cancers 31. TOP2A contributes to oxaliplatin-resistant patients with metastatic colorectal cancer 32. Recently, Cai *et al* found that the high expression of TOP2A is corresponding to the progression and poor prognosis of HCC by bioinformatic studies, but the specific mechanism of TOP2A in HCC is not elucidated. Here, we confirmed that TOP2A was upregulated in HCC tissues and was related to T stage, M stage and poor prognosis of HCC. Furthermore, we found that TOP2A enhanced proliferation, migration and invasion capabilities of HCC cells both *in vitro* and *in vivo*. These results indicated that TOP2A played a role in HCC progress.

Cell cycle regulation is strongly significant in the development of magnificence. In this study, GSEA analysis showed that high expression of TOP2A was closely associated with cell cycle. Fluorescence activated Cell Sorting (FASC) showed that TOP2A knockdown could inhibit cell proliferation by inducing cell cycle arrest from G2 to M phase, which is consistent with previous reports 20. In this pathway CDK1-Cyclin B1 complex plays an important role in promoting the G2/M phase transition. Particularly, CHK1 phosphorylation suppresses the formation of CDK1-CyclinB1 complex and blocks the progression of the cell cycle 21-24. We found that knockdown and overexpression of TOP2A respectively promotes and inhibits the phosphorylation of CHK1. The TOP2A expression varies with the expression level of CDK1 and Cyclin B1.

Metastasis is a multi-step process in which tumor cells leave the primary tumor and spread to a distant location, which is a key factor affecting the survival rate of tumor patients. EMT is commonly considered as an important mechanism of migration and invasion for most cancer cells. EMT makes cells lose the ability of cell-to-cell adhesion but gain the ability of migration and invasion, which contribute to the wound healing and tumor metastasis. In the process of EMT, the expression of epithelial molecules, E-cadherin for example, decreased, while the expression of interstitial molecules such as N-cadherin, vimentin and Snail increased. In our study, we demonstrated that knocking down TOP2A inhibited HCC cell migration and invasion *in vitro*, and inhibited tumor growth and metastasis *in vivo*, downregulated E-cadherin and upregulated N-cadherin, Vimentin and Slug was observed in HCC cells overexpressing TOP2A. On the contrary, knocking down TOP2A produced opposite results. These data suggest that TOP2A can promote the metastasis of HCC cells by inducing EMT.

To explore the regulation mechanism of TOP2A, we found that TOP2A can be the target of miR-144-3p. miRNA is an endogenous non-coding protein RNA gene with approximately 18-24 nucleotides in length. It is known to have effects on a variety of diseases and to regulate the production of proteins by interacting with mRNA 16, 33, 34. Previous studies have shown that miR-144-3p can regulate the proliferation, migration, invasion and biological behaviors of various tumor cells that are drug resistant 35-37. For example, miR-144-3p promotes the tumor growth and metastasis of papillary thyroid carcinoma by targeting paired box gene 8 38. miR-144-3p regulates the resistance of lung cancer to cisplatin by targeting Nrf2 39. Another group found that miR-144-3p is down-regulated and inhibits the progress of HCC 40. All these researches above did not elucidate the specific mechanism 41-43. In this study, using the dual luciferase reporter assay we verified that TOP2A was a direct target of miR-144-3p that suppressed TOP2A expression in HCC cells. The expression of miR-144-3p and TOP2A was negatively correlated in HCC cells. Overexpression of miR-144-3p resisted the process that TOP2A promoted the proliferation, migration, invasion and EMT of hepatocellular carcinoma cells. Together, we propose that TOP2A is negatively regulated by miR-144-3p and affects the progression of hepatocellular carcinoma.

In summary, our study reports that TOP2A is up-regulated in HCC tissue and is related to poor prognosis. In details, TOP2A enhances the proliferation, migration and invasion capabilities of HCC cells *in vitro* and *in vivo*. It also promotes the transition of cell cycle from G2 phase to M phase and the EMT process, thus leading to the progress of HCC. In addition, our data suggest that overexpression of miR-144-3p can resist the process that TOP2A promotes the proliferation, migration invasion of hepatocellular carcinoma cells. The exploration of TOP2A provides a novel perspective into the mechanism of HCC.

## Supplementary Material

Supplementary figure and table.

## Figures and Tables

**Table 1:** TOP2A expression in HCC and clinicopathological characteristics (n=90).

TOP2A expression			
	Low (n=41)	High (n=49)	P value
Gender			0.765
Male	36	44	
Female	5	5	
Age (years)			<0.001
≤ 60	5	8	
>60	36	41	
Tumor grade			0.004
Well	13	3	
Moderate	27	41	
Poor	1	5	
Tumor T stage			0.007
T1	17	6	
T2	14	24	
T3, T4	10	19	
Tumor N stage			0.544
N0	41	48	
N1	0	1	
Tumor M stage			0.018
M0	35	31	
M1	6	18	
Tumor stage			<0.001
Ⅰ	19	2	
Ⅱ	8	13	
Ⅲ	8	16	
Ⅳ	6	18	
