# Supplementary material for: TOP2A promotes proliferation and metastasis of hepatocellular carcinoma regulated by miR-144-3p
Source: J Cancer. 2022 Jan 1;13(2):589–601. doi: 10.7150/jca.64017 (PMC8771514; doi:10.7150/jca.64017)
Supplement: Supplementary file 1 — Supplementary figure and table. [file jcav13p0589s1.pdf]

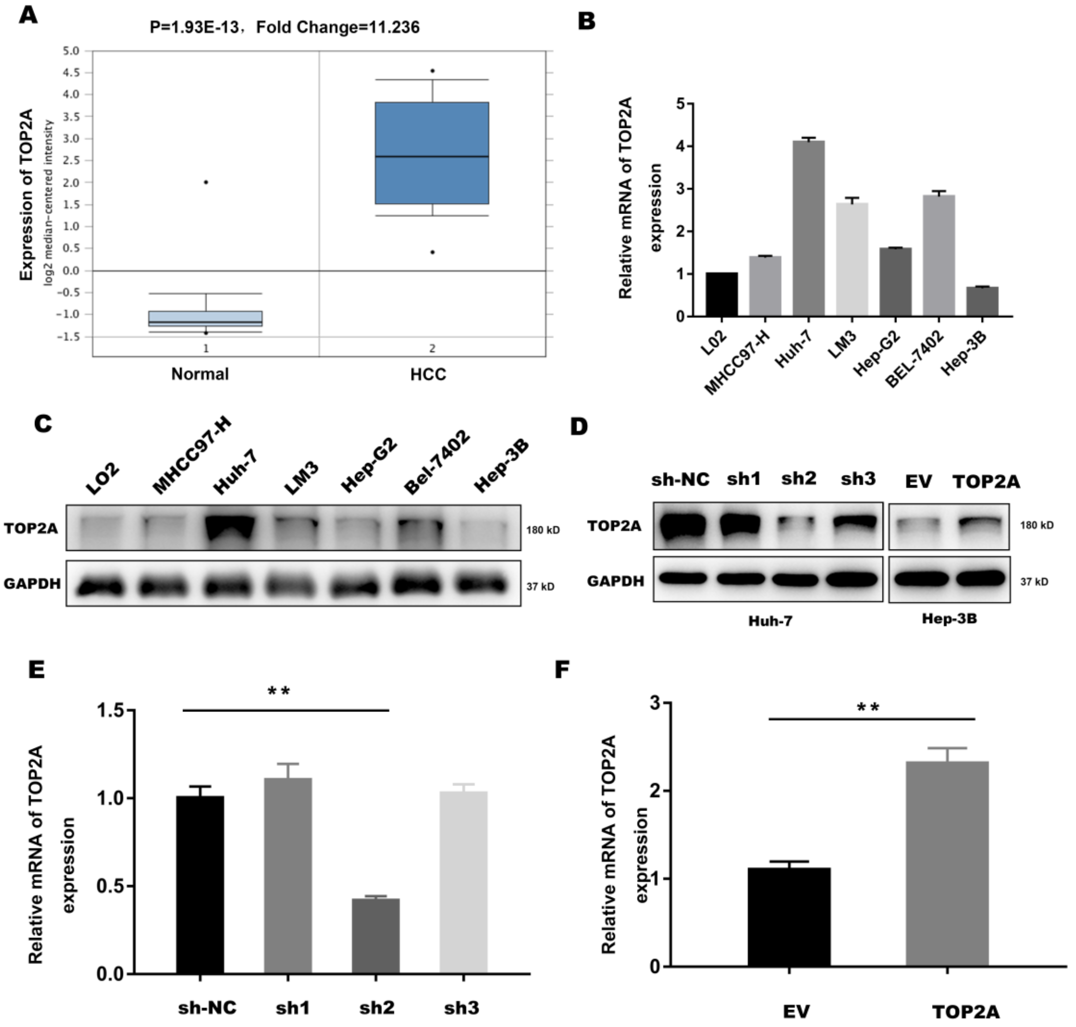

Figure S1

(A) The mRNA expression level of TOP2A in HCC tissues and normal tissues from the ONCOMINE database. **(B, C)** The mRNA and protein expression level of TOP2A in a human normal LO2 cell and 6 HCC cell lines. **(D, E, F)** Western blot and RT-qPCR detection of TOP2A expression after lentiviral expression of sh-TOP2A and TOP2A-overexpressing. ** $P < 0.01$

Table. S1

	Primers	Sequence (5’ to 3’)
1	GAPDH-F	TGTGTCCGTCGTGGATCTGA
	GAPDH-R	CCTGCTTCACCACCTTCTTGA
2	TOP2A-F	TTCTTGATATGCCCCTTTGG
	TOP2A-R	GCTTCAACAGCCTCCAATTC
3	miR-144-3p-F	GCGCGCGTACAGTATAGATGA
	miR-144-3p -R	AGTGCAGGGTCCGAGGTATT
	miR-144-3p-RT	GTCGTATCCAGTGCAGGGTCCGAGG
		TATTCGCACTGGATACGACAGTACA
4	hsa-U6-F	GCTCGCTTCGGCAGCACATATAC
	hsa-U6-R	AGTGCAGGGTCCGAGGTATT
	has-U6-RT	GTCGTATCCAGTGCAGGGTCCGAGGTAT
		TCGCACTGGATACGACAAAATATGG
